# Analysis of Aesthetic Preferences Regarding Gingival–Dental Color Combinations

**DOI:** 10.1111/jerd.13498

**Published:** 2025-06-10

**Authors:** Cristina Gómez Polo, Ana María Martín Casado

**Affiliations:** ^1^ Department of Surgery, School of Medicine University of Salamanca Salamanca Spain; ^2^ Department of Statistics, School of Medicine University of Salamanca Salamanca Spain

## Abstract

**Statement of Problem:**

It is our view that the color of teeth and gingiva needs to be analyzed conjointly, given their close anatomical interrelation and the lack of research on perceptions of this chromatic combination.

**Objective:**

To determine aesthetic preferences concerning the chromatic combination of ceramic gingival specimens and acrylic teeth and analyze the influence of age and sex.

**Material and Methods:**

A sample of 120 participants responded to a survey, in which each participant first selected the three “ad hoc” ceramic gingival specimens whose color they considered the most attractive from seven Vita Lumex AC Gingiva colors (231–237). Each participant then allocated a score from 1 to 10 for the chromatic combination of each of the three ceramic gingival specimens chosen in combination with each of the three acrylic teeth (maxillary central incisor, maxillary lateral incisor, and maxillary canine) in the three most frequent colors in the reference population (1M1, 3M1, and 2L1.5). The questionnaire also collected data on age and sex. Statistical analysis of the results was conducted using SPSS (v.28) software.

**Results:**

Participants most frequently chose gingival shade 232 as their first preference (80.8%), shade 233 was most frequently placed in second position (75%), and most participants selected shade 235 as their third preference (58.3%). The highest rated gingiva‐tooth shade combination was 232‐1M1 (mean score 7.9), followed by 232‐3M1 (mean score 7.3). The only significant difference (*p* < 0.05) between men and women was between the mean scores allocated for the 232‐3M1 combination, which was rated higher by women than men. Statistically significant differences were found between the mean scores allocated by different age groups for the 233‐3M1, 233‐2L1.5, and 235‐1M1 combinations. Younger participants scored the first two shade combinations lower, while the opposite was true for the last combination.

**Conclusions:**

When establishing aesthetic preferences, gingival color takes priority over dental color when both structures are visible in combination. Preferences regarding the most attractive gingival shades are focused on only two colors (232 and 233). A substantial majority of participants rated the 232‐1M1 gingival–dental shade combination most highly. Practically no significant differences were identified between the mean scores allocated for gingival–dental color combinations according to sex. Age made a greater difference to results, although both variables had only a modest impact.

**Clinical Significance:**

Producing a combined gingival–dental shade guide would be useful, enabling the patient to provide their complete aesthetic vision, particularly in clinical situations where gingival tissue needs to be restored in patients with high smile lines. Manufacturers need to improve their gingival shade ranges, due to the limited number available, their disparity with natural gingival color, and the fact that patients do not like most shades on offer. A large percentage of aesthetic preferences focused on a limited number of gingival–dental color combinations, with lighter dental shades combined with the gingival shades that best approximate natural gingival color considered the most aesthetic. Age has more influence on gingival–dental color preferences than sex, although both factors have only a modest impact. For high smile lines, patients prioritize gingival color within the gingival–dental chromatic combination, since the preference for a certain shade of gingival specimen is maintained, irrespective of tooth color, indicating a stable chromatic hierarchy.

In a social context in which aesthetics has a significant impact, dentistry has advanced by focusing its efforts not only on masticatory function, but on achieving more beautiful, natural‐looking treatment results [1, 2]. The advances made with dental prosthetic rehabilitation have been numerous, with the incorporation of a greater variety and number of materials, starting with feldspathic ceramics, their subsequent development with porcelain fused to metal crowns in 1960, followed by more recent materials such as zirconia, lithium disilicate, and current ceramics [1, 2, 3, 4, 5]. While there have been numerous improvements in dental prosthetic materials, primarily focused on teeth and “white aesthetics,” less attention has been paid to “pink aesthetics” (gingiva). This has led to a limited range of standardized gingival shades for fixed or removable prostheses [6, 7, 8, 9]. The variability in natural gingival color, influenced by factors like ethnicity, vascularization, and pigmentation, exacerbates this issue [6, 10, 11, 12]. The few physical gingival shade guides available do not cover the entirety of this natural gingival color space, and are lacking published data on the methodology used in their chromatic design, the standard shades on the market apparently having been produced without adherence to any specific criteria [6, 7, 8, 9, 10, 11, 12]. Further, distinct manufacturers offer basic pink shades that share the same name but are chromatically very different [8]. Without a comprehensive, standardized gingival shade guide, clinicians must rely on artistry, complicating the achievement of optimal aesthetic results in prosthetic and direct restorations [12].

Studies emphasize that achieving a beautiful smile requires both positive dental and gingival aesthetics [8, 9, 13, 14]. Color plays a crucial role in patient satisfaction with prosthetic rehabilitations [8, 13, 15], and 80% of people show gingiva when they smile [16]. Displaying maxillary gingiva is associated with youthfulness, and aesthetic facial treatments may increase its prevalence, making gingival aesthetics more important throughout life.

Advancements in color selection technology, including spectrophotometers and colorimeters using the CIELAB system, have reduced errors compared to subjective methods [15, 17, 18, 19, 20, 21]. The CIELAB system, established in 1976 by the International Commission on Illumination (CIE) [22], quantifies color using coordinates and enables precise comparisons with the Euclidean formula (Δ*E*_ab_*) [23, 24, 25]. Improved color‐difference formulae, like the CIEDE2000 formula (Δ*E*_00_) [26], better align with human vision and are suitable for clinical studies [27, 28, 29]. The ability to quantify color and advances in this field opened up research on perceptibility thresholds (PT) and clinical acceptability thresholds (AT) to help assess color differences in dentistry [9, 21, 30, 31]. The various studies conducted offer ranges of values for these thresholds in the dental color space (AT from 2.72 to 6.8 using Δ*E*_*ab*_) [32, 33] and the gingival color space (AT from 4.1 to 4.6 using Δ*E*_*ab*_) [13, 34].

Subjective color selection using physical shade guides remains widely used in clinical practice [17, 18, 35, 36] despite its imprecision, influenced by factors like observer‐related parameters and ambient conditions [9, 13, 17, 36, 37, 38]. Improved shade guides are needed to offer greater precision and aesthetically pleasing options for dental and gingival color spaces [39].

Gingival aesthetics has been less studied compared to dental aesthetics, with limited research on natural or inflamed gingival color, the degree of accuracy achieved in selective color selection [40], training improvements, or new shade guide proposals [9, 41]. Additionally, much of the published research has small sample sizes or provides low levels of evidence [7, 10, 42, 43, 44]. All of the aforementioned studies analyze either dental color [45, 46, 47] or gingival color [6, 12, 34, 41, 42] but research on chromatic perceptions and preferences regarding both viewed simultaneously is lacking.

Although restoring the gingiva with prostheses is becoming increasingly important [6, 11, 14, 48], there are no studies analyzing aesthetic preferences when people can evaluate combinations of dental and gingival shades at the same time. Choosing these shades separately can give a less accurate idea of the final color. Since 1839, it has been known that colors of objects seen together appear more different than when seen separately [49], meaning the colors of gingiva and teeth are perceived as part of the same visual field. Understanding how pink and white shade combinations affect patient perceptions could improve subjective color selection and help develop better tools for aesthetic decisions. It would be clinically useful for this study to start a line of research on patient perceptions of pink–white shades viewed in juxtaposition, with the ultimate aim of developing a combined shade guide in which the gingival (pink) and dental (white) aesthetic options appear together. This could serve as a reference tool, particularly for totally edentulous patients or in cases where both tissues need to be restored and reproducing the color of natural adjacent tissues is not necessary.

The present research has the following objectives and null hypotheses. Objective 1: Describe the aesthetic preferences of participants regarding the gingival‐dental chromatic combinations for prostheses. Null hypothesis 1: No significant differences exist between the aesthetic preferences of participants for the distinct gingival–dental combinations. Objective 2: Analyze whether there are differences relating to sex and age between the aesthetic preferences of participants regarding the distinct gingival–dental combinations. Null hypothesis 2: No significant sex‐ or age‐related differences exist between aesthetic preferences for the distinct gingival–dental combinations evaluated.

## 1 Material and Methods

### 1.1 Material

**FIGURE 1:**
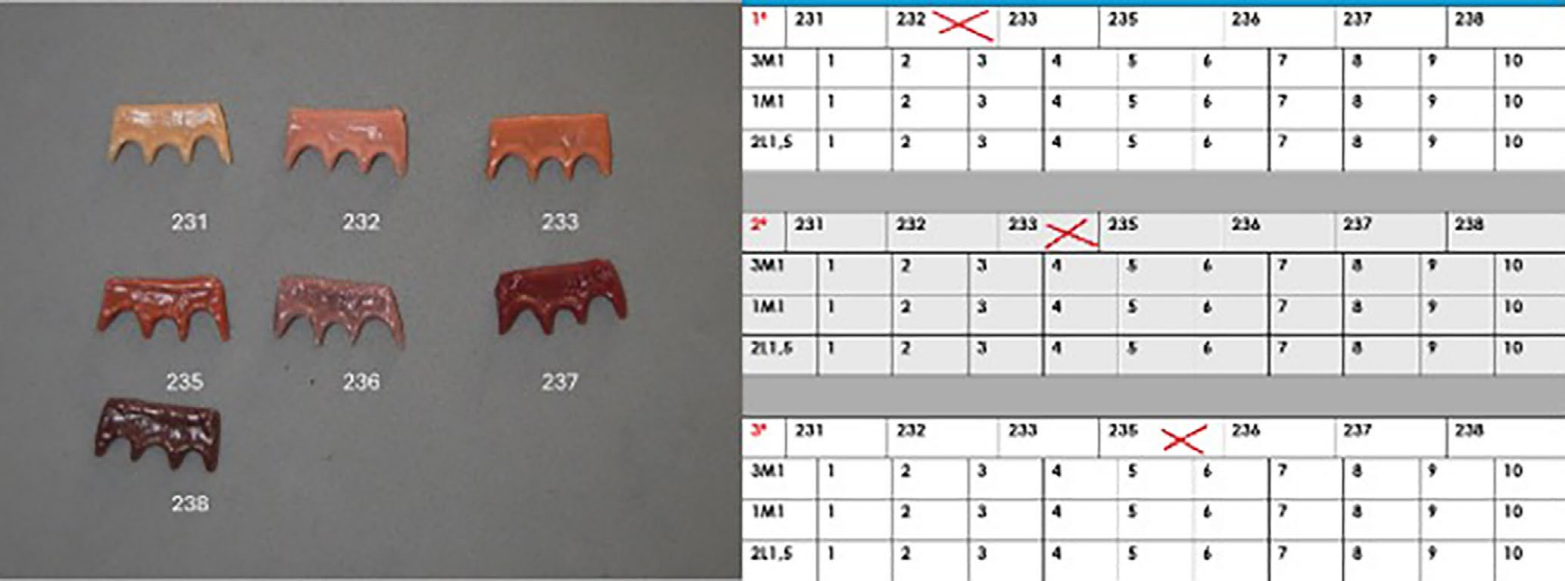
Selection of gingival preferences and an example of a participant's selection and ranking.

**FIGURE 2:**
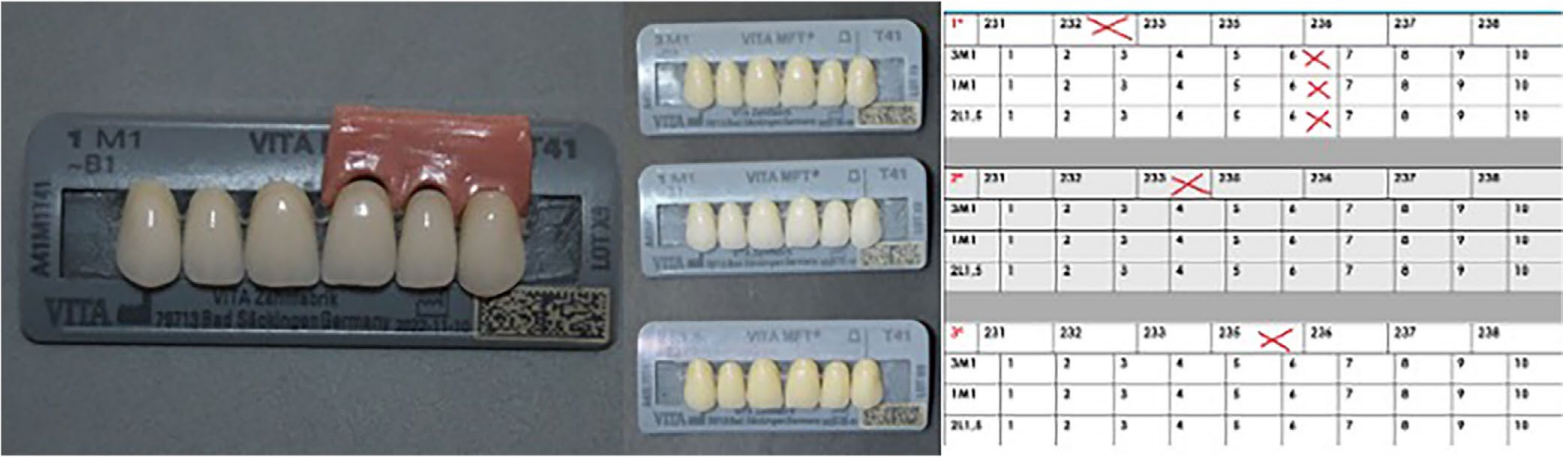
Example of a gingival specimen combined with Vita MFT maxillary anterior acrylic teeth, model T41 (triangular‐shaped) in shades 3M1/D2, 1M1/B1, and 2L1.5/B2, with an example of how combinations were scored.

The first step was to produce festooned gingival specimens for the maxillary aesthetic zone (Figure 1), using Vita Lumex AC Gingiva (Vita Zahnfabrik) ceramics, following the manufacturer's instructions. All available gingival shades were used: pale papilla 231 (ref: S93990; batch: 16D20230516Q), light rose 232 (ref: S89040; batch: 16D20230727M), nectarine 233 (ref: S96110; batch: 16D20230313A), rosewood 235 (ref: S94010; batch: 16D20230615E), purple 236 (ref: S98230; batch: 16D20230502I), deep red 237 (ref: S89690; batch: 16D20230829+), and dark red 238 (ref: S98100; batch: 16D20230227K). The design included three anatomical areas: the interdental papillae, free gingival margin, and attached gingiva. The ceramic gingival specimens were festooned to fit with the shape of VITA MFT (Vita Zahnfabrik) maxillary anterior acrylic teeth, model T41 (triangular‐shaped), in the following dental colors: 3M1/D2 (ref: A43M1T41; batch: T9), 1M1/B1 (ref: A41M1T41; Lot X9), and 2L1.5/B2 (ref: A42L15T41; batch: G9) (Figure 2). After firing all the ceramic gingival specimens, the recommended polishing and glazing steps were completed.

To measure the color coordinates of the ceramic gingival specimens and acrylic teeth, a SpectroShade Micro spectrophotometer (MHT, Zürich, Switzerland) was used. Color measurement of all specimens was performed with the same fluorescent zenithal lighting (TLD 95/65) and at the same time in the morning, against a neutral gray background. The color coordinates of the gingival samples and the acrylic teeth were recorded in the middle part. The spectrophotometer was calibrated according to the manufacturer's instructions before each measurement, and color coordinates were measured three times for each artificial tooth and each gingival specimen (Table 1).

**TABLE 1:** Mean CIELAB color coordinates for dental and gingival shades used.

		L*	a*	b*
Ceramic gingival shades	231	69.2	10.5	27.2
	232	60.0	21.9	16.2
	233	55.3	24.7	24.9
	235	42.3	27.7	19.6
	236	46.0	18.1	7.0
	237	33.6	27.4	11.1
	238	32.5	11.9	7.6
Dental shades	3M1	71.2	0.8	15.8
	1M1	77.2	−0.1	11.9
	2L1.5	73.5	0.4	19.5

Once these measurements had been made, the color differences between the distinct gingival shades were calculated, using the Δ*E*_*ab*_ formula and Δ*E*_00_ formula. The color coordinates of the ceramic gingival specimens fell within the following ranges. L*: 32.5–69.2; a*: 21.1–69.1; and b*: 7.0–27.2. The color differences between the ceramic gingival specimens ranged from 10.22 to 42.53 units, according to the CIELAB Euclidean formula, and 6.40–39.84 units, according to the CIEDE2000 formula. The color differences between the three dental shades used ranged from 4.35 to 8.48 units, according to Δ*E*_*ab*_ formula, and 2.73–5.25 units, according to Δ*E*_00_ formula. All values exceed the clinical acceptability thresholds (AT) for the gingiva [13, 34] and teeth [32, 33].

The researchers also calculated the color differences between the gingival shades used and natural gingival color in the middle zone of participants from a previous study [10], using both formulae (Table 2). This data shows the ceramic gingival shades that are most similar to the color of the middle zone of natural attached gingiva [10] to be shades 232, 235, and 233.

**TABLE 2:** Mean ± SD of color differences between each ceramic gingival specimen and the middle zone of attached gingiva from a prior in vivo database [10].

Ceramic gingival shade	Δ*E*_*ab*_ ± SD	Δ*E*_00_ ± SD
231	26.46 ± 6.00	22.36 ± 5.58
232	11.10 ± 5.41	9.90 ± 5.29
233	12.63 ± 3.77	9.09 ± 3.87
235	11.83 ± 4.27	9.76 ± 4.37
236	13.05 ± 3.62	8.90 ± 3.22
237	18.42 ± 5.66	16.31 ± 5.83
238	24.00 ± 4.09	18.76 ± 4.71

## 2 Methodology

### 2.1 Data Collection

The aesthetic preferences of a sample of 120 participants were recorded, including 74 women (61.7%) and 46 men (38.3%). Inclusion criteria were being aged between 18 and 80 years and lacking visual or cognitive disorders. All participants included in the study passed the Ishihara test. Participant ages ranged from 18 to 69 years, with a mean and standard deviation of 35.7 and 14.2. The distribution by age group was as follows: 41 participants (34.2%) aged between 18 and 25, 51 participants (42.5%) between 26 and 50, and 28 participants (23.3%) aged over 50. The present study was approved by the ethics committee of the University of Salamanca (Spain). Participants voluntarily accepted to take part in the study and completed a questionnaire on (1) age and sex, (2) preferences regarding gingival aesthetics, and (3) preferences regarding combined gingival–dental aesthetics.

Analysis of participants' visual aesthetic preferences was conducted in a dental clinic with ambient lighting from a Philips TLD 95 fluorescent lamp, which emits light ranging from 5000 to 5500 K. This color temperature is similar to natural daylight, which is important to ensure precise shade selection. In order to optimize color discrimination and increase visual acuity, each participant placed the samples at the distance of their choice.

The gingival specimens were combined with anterior acrylic teeth cards (13–23) in dental shades corresponding to 3M1, 1M1, and 2L1.5 from the Vita 3D Master guide. These shades were chosen as the most common in the population under study [50]. The set of physical gingival specimens used in the present research were designed and produced “ad hoc,” following a two‐stage process. (1) In the first stage, participants were asked to choose the three individual gingival specimens they liked most from the seven available and rank them in order of preference in the table, using the numbered boxes provided (1st, 2nd, and 3rd) (Figure 1). The specimens were identified using the numeric code assigned by Vita (231, 232, 233, 235, 236, 237, and 238). (2) In the second stage, participants were asked to score each of the three gingival specimens chosen when combined with the artificial anterior teeth (maxillary central incisor, maxillary lateral incisor, and maxillary canine), in the three most common shades for artificial teeth in the study population [50]: 3M1, 1M1, and 2L1.5, from the Vita 3D Master shade guide. Scores of 1–10 were given, marking the score assigned to each gingival–dental combination on the rows corresponding to each dental shade (3M1, 1M1, and 2L1.5) (Figure 2).

### 2.2 Statistical Analysis

Tables of frequencies and percentages were used to describe the data collected on preferences in the first stage, while tables containing mean values and standard deviations were used to describe the preferences recorded in the second stage. Comparison of the mean scores for the three gingival–dental combinations for each gingival shade was performed using the one‐way ANOVA with a randomized block design (participants), while the mean scores for the five gingival–dental combinations for each dental shade were compared using the one‐way ANOVA with a completely randomized design. Effect sizes were assessed using Cohen's d and eta squared [51, 52]. The statistical significance level was set at 0.05, and SPSS (v.28) was used to perform all analyses.

## 3 Results

### 3.1 Description of Aesthetic Preferences Regarding the “Ad Hoc” Ceramic Gingival Specimens and Artificial Teeth

Stage 1 Selection of the three most attractive shades from the ceramic gingival specimens.

Table 3 shows the number of participants that selected each gingival specimen as one of their three choices, and the order of preference. Most participants (97 out of 120) selected shade 232 as their first choice, while 233 was the second most preferred shade (90 out of 120). Shades 232 and 233 were chosen in one of the three positions, in most cases first and second, by 118 (98.3%) and 113 (94.2%) participants, respectively. The next most preferred shade was 235, which was placed in one of the three positions, albeit mainly third, by 77 participants (64.2%).

Stage 2 Scores (1–10) allocated for the aesthetic combination of the three ceramic gingival specimens chosen with the three most common shades of acrylic artificial teeth in the study population.

**TABLE 3:** Frequency of selection (percentage, %) of gingival shades, according to order of preference.

Shade of ceramic gingival specimen	*n* (percentage, %)		
	First	Second	Third
231	1 (0.8)	3 (2.5)	25 (20.8)
232	97 (80.8)	17 (14.2)	4 (3.3)
233	17 (14.2)	90 (75.0)	6 (5.0)
235	2 (1.7)	5 (4.2)	70 (58.3)
236	1 (0.8)	3 (2.5)	9 (7.5)
237	1 (0.8)	2 (1.7)	4 (3.3)
238	1 (0.8)	0 (0.0)	2 (1.7)

Table 4 shows the mean values and standard deviations for the scores allocated to each combination of ceramic gingiva–acrylic artificial teeth. As the data shows, the number of participants who scored each combination is not constant, due to the data collection process described above. Table 4 also provides the *p* values obtained in the comparison of the mean scores for the three gingival–dental combinations for each gingival shade (last column) and the *p* values from the comparisons of the mean scores for the five gingival–dental combinations for each dental shade (bottom row). Only the five most representative gingival shades appear in Table 4, since shades 237 and 238 were chosen a negligible number of times, as shown in Table 2. Of the combinations with gingival shade 232, the dental shade with the highest mean score is 1M1, which also scored highest in combinations with gingival shade 233, although the mean score for 232‐1M1 is higher than that for 233‐1M1. Of the dental shades chosen in combination with gingival shade 235, the highest mean score was for 3M1, although this is considerably lower, at only 5.00, than the aforementioned mean scores. The three highest mean scores were secured by gingival shade 232 in combination with all three dental shades. It is worth noting the low mean scores for some combinations, including the three scores for combinations containing gingival shade 231 and all three containing gingival shade 236, all of which were below 5.00. This indicates that participants did not consider the shades of the anterior teeth selected for this study to be the most aesthetically pleasing for those gingival shades.

**TABLE 4:** Mean values (SD) of scores (1–10) for most frequently selected combinations of ceramic gingival specimen–artificial teeth, with *p* values of tests comparing scores.

Shade of ceramic gingival specimen	*n*	Dental shade			*p*
		3M1	1M1	2L1.5	
231	29	4.14 (2.57)	4.52 (2.26)	3.93 (2.31)	0.106
232	118	7.31 (1.59)	7.92 (1.53)	6.89 (1.39)	**< 0.001**
233	113	6.27 (1.46)	6.69 (1.54)	6.24 (1.68)	**0.013**
235	77	5.00 (1.76)	4.84 (2.00)	4.94 (2.00)	0.662
236	13	3.92 (2.63)	4.38 (2.69)	4.00 (2.68)	0.751
*p* value		**< 0.001**	**< 0.001**	**< 0.001**	

*Note:* Statistically significant *p* value are marked in bold.

### 3.2 Differences Between Aesthetic Preferences for the Combined Prostheses (Ceramic Gingival Specimens and Acrylic Artificial Teeth) in Relation to Sex and Age

**FIGURE 3:**
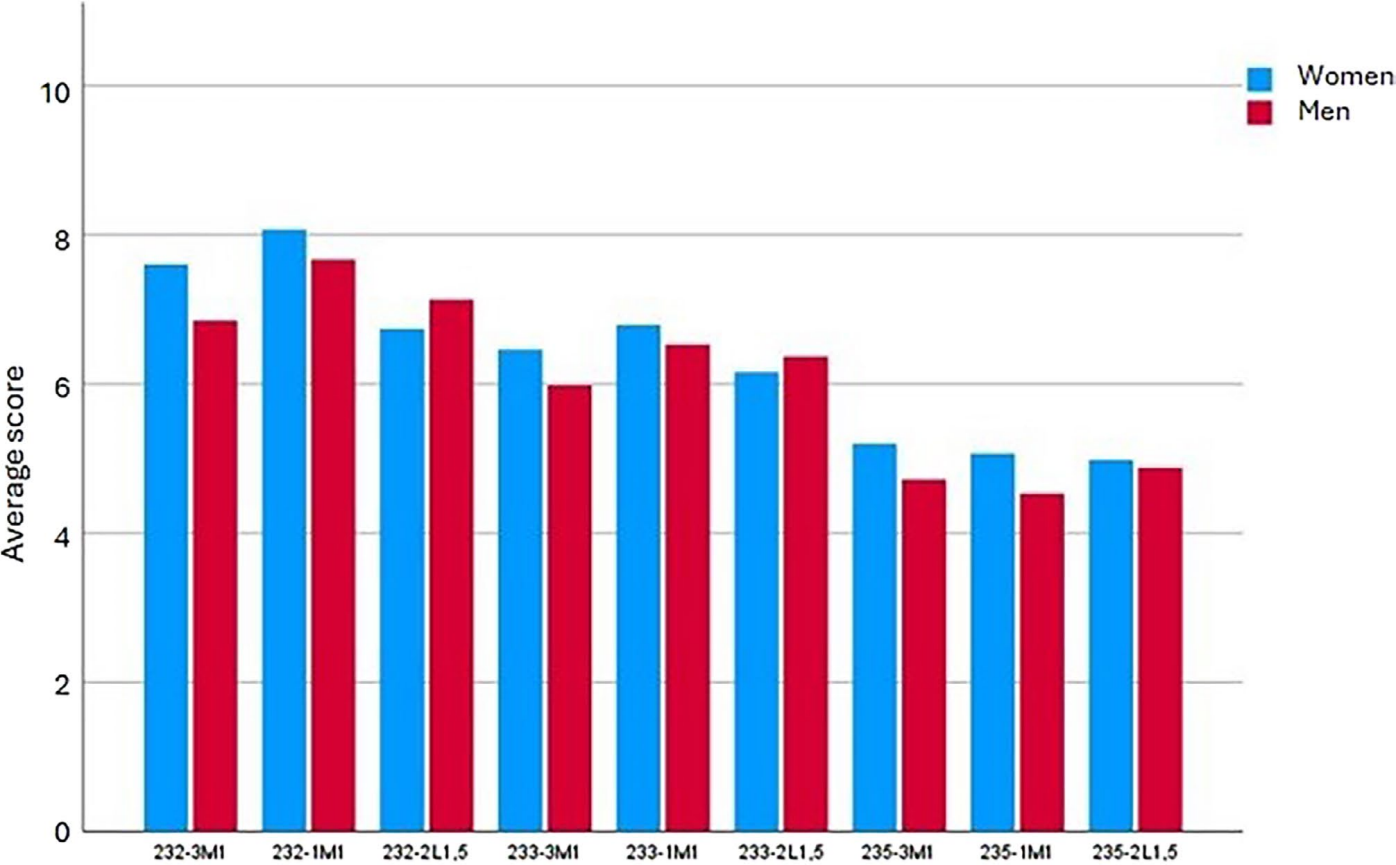
Mean scores, according to sex, for highest‐rated (1–10) combinations of ceramic gingival specimens and artificial teeth.

Only the gingival shades that participants rated most highly (232, 233, and 235) were used in the analysis of sex‐ and age‐related differences between preferences for the ceramic gingival specimen–artificial teeth combinations. Table 5 shows the mean values and standard deviations (SD), as well as the statistics, *p* values, and effect sizes for the comparisons made, in relation to sex. The only significant difference (0.01 < *p* < 0.05) was found when comparing the mean scores given by men and women for the 232‐3M1 combination. Women scored this gingival–dental color combination higher than men, albeit with a small to moderate effect size [52] (Figure 3). The comparisons of scores for all other combinations according to sex produced differences that were not significant. Table 6 shows the mean values and standard deviations, as well as the statistics, *p* values, and effect sizes for the comparisons made, in relation to age group.

**TABLE 5:** Results obtained in the comparison of mean scores for ceramic gingival specimen (231, 232, 233, 235, 236, 237, and 238 gingival shades)–artificial teeth combinations, in relation to sex.

	*n*	Mean	SD	*t*	*p*	Cohen's *d*	Effect size
232‐3M1				2.559	**0.012**	0.483	Small–moderate
Women	72	7.60	1.50				
Men	46	6.85	1.63				
232‐1M1				1.377	0.171	0.260	Small
Women	72	8.07	1.68				
Men	46	7.67	1.23				
232‐2L1.5				−1.513	0.133	−0.286	Small
Women	72	6.74	1.42				
Men	46	7.13	1.31				
233‐3M1				1.706	0.091	0.331	Small
Women	70	6.46	1.39				
Men	43	5.98	1.55				
233‐1M1				0.842	0.402	0.163	None
Women	70	6.79	1.61				
Men	43	6.53	1.40				
233‐2L1.5				−0.660	0.510	−0.128	None
Women	70	6.16	1.63				
Men	43	6.37	1.76				
235‐3M1				1.184	0.240	0.274	Small
Women	45	5.20	1.93				
Men	32	4.72	1.49				
235‐1M1				1.160	0.250	0.268	Small
Women	45	5.07	2.05				
Men	32	4.53	1.92				
235‐2L1.5				0.235	0.815	0.051	None
Women	45	4.98	2.26				
Men	32	4.88	1.58				

*Note:* Statistically significant *p* value are marked in bold.

**TABLE 6:** Results obtained in comparison of mean scores (1–10) for the ceramic gingival specimen–artificial teeth combinations, allocated by participants in three age groups.

	*n*	Mean	SD	*F*	*p*	*η* ^2^	Effect size
232‐3M1				1.663	0.194	0.028	Small
18–25 years	41	7.56	1.83				
26–50 years	50	7.34	1.27				
Over 50 years	27	6.85	1.68				
232‐1M1				0.694	0.502	0.012	None
18–25 years	41	7.93	1.46				
26–50 years	50	8.06	1.39				
Over 50 years	27	7.63	1.86				
232‐2L1.5				0.565	0.570	0.010	None
18–25 years	41	6.95	1.41				
26–50 years	50	6.74	1.40				
Over 50 years	27	7.07	1.36				
233‐3M1				4.940	**0.009**	0.082	Moderate
18–25 years	36	5.67	1.49				
26–50 years	49	6.51	1.29				
Over 50 years	28	6.64	1.52				
233‐1M1				0.768	0.466	0.014	None
18–25 years	36	6.56	1.18				
26–50 years	49	6.61	1.47				
Over 50 years	28	7.00	2.00				
233‐2L1.5				5.531	**0.005**	0.091	Moderate
18–25 years	36	5.53	1.76				
26–50 years	49	6.45	1.63				
Over 50 years	28	6.79	1.34				
235‐3M1				3.039	0.054	0.076	Moderate
18–25 years	24	5.71	1.60				
26–50 years	35	4.74	1.80				
Over 50 years	18	4.56	1.69				
235‐1M1				5.465	**0.006**	0.129	Moderate
18–25 years	24	5.75	1.85				
26–50 years	35	4.11	1.84				
Over 50 years	18	5.06	2.04				
235‐2L1.5				1.419	0.248	0.037	Small
18–25 years	24	5.50	1.86				
26–50 years	35	4.66	1.96				
Over 50 years	18	4.72	2.19				

*Note:* Statistically significant *p* value are marked in bold.

**FIGURE 4:**
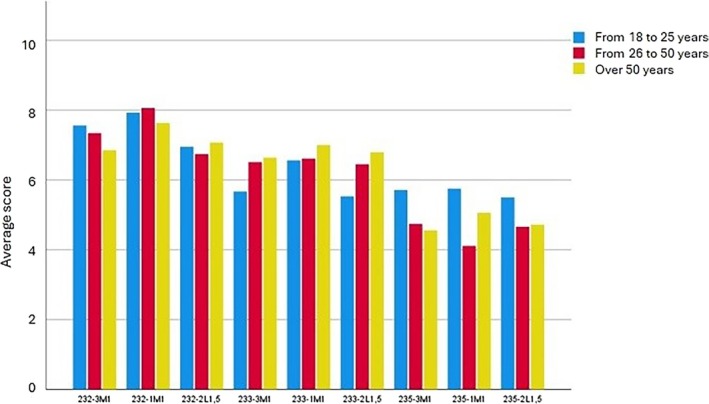
Mean scores, according to age group, for highest‐rated (0–10) combinations of ceramic gingival specimens and artificial teeth.

Statistically significant differences between the mean scores allocated by different age groups were obtained for 233‐3M1, 233‐2L1.5, and 235‐1M1: for the first two combinations, younger participants gave lower scores than older participants, while the reverse was true for the third combination (Figure 4). The effect sizes were moderate in all three cases. The other comparisons did not produce significant results, although the comparison of scores for the 235‐3M1 combination is worth highlighting, where differences came very close to statistical significance. The distinct effect sizes of the comparisons according to sex and age group show that age influences preferences more than sex, although the impact of both factors is relatively modest.

## 4 Discussion

The main limitation of this research is the use of ceramic and acrylic materials when others are available with different surface properties that could create distinct perceptive experiences. While gingival porcelain is the most frequently used material for indirect fixed restorations, offering a natural, aesthetic appearance, long‐term chromatic stability, and good performance in reproducing the color of distinct anatomical regions, other options are viable in certain clinical situations [11, 53]. For example, gingival recessions can be treated with periodontal surgery or direct restorations using pink resin composites. The chromatic variety of gingival resin composites available is also limited [54, 55], but can be directly expanded in the clinic using opaquers or color modifiers, which is an advantage not offered by ceramics. Another limitation is the decision not to show participants all 21 possible gingival specimen–acrylic teeth combinations, but the researchers decided against this due to the increased observation time needed and the resulting visual fatigue. It is important to keep factors related to human observation in mind, which are not quantifiable using survey methodology but may influence responses, including tiredness, each participant's level of interest in the study objectives, prior dental chromatic experiences, personality, and profession. When choosing among the three colors of acrylic teeth used, it should be taken into consideration that this selection is limited to the most frequent colors in the Spanish [50] population and may differ in other populations.

Research determining the frequency of dental colors in different national settings is relatively scarce, and results differ in geographically and culturally diverse populations, such as New York state [56], Turkey [57], India [58], and Spain [59], which is what motivated the authors to focus on the Spanish population, in which the most common shade, according to Gómez‐Polo et al. [50], referring to the Vita 3D Master guide, is 3M1, followed by 1M1.5 and 2L1.5. Given that 1M1.5 is not commercially available for acrylic teeth, 1M1 was chosen for this study, as the shade that most closely resembles it.

The novelty of examining aesthetic preferences regarding the color of combined gingival–dental prostheses prevents direct comparison with other studies, since prior research on this topic is lacking. It is worth noting that the vast majority of studies on perception use a single tooth or gingival specimen, while the use of festooned specimens in the present study entailed combining them with a maxillary central incisor, lateral incisor, and canine, creating a visual experience that better approximates clinical reality.

To determine how close the ceramic gingival shades analyzed are to natural Caucasian gingival color [6], the differences were calculated (Table 2) between these ceramic gingival shades and the natural color of Caucasian subjects in a pre‐existing database [6] (color coordinate ranges for the healthy gingival color space—L*: 28.3–65.4; a*: 11.1–37.2; b*: 6.9–25.2). The greatest mean color difference was found for shade 231 (the lightest), showing its lack of similarity to natural gingival color, added to which its color coordinates fall outside the coordinate range of the healthy gingival color space. This may explain why it was not among the most preferred shades of the present study's participants. This shows that preferences regarding gingival color do not reflect dental color preferences, where lighter teeth are preferred by the population, even if outside the natural color space. Participants did not prefer overly light gingival shades, as shown by the negligible percentage who chose gingival shade 231 as the most attractive (0.8%). In contrast, gingival shade 232 is the closest to natural healthy gingival color (Table 2) and all of its coordinates fall within the healthy gingival color space [6], which may explain why it was chosen in first position most often (80.8%). Additionally, the combinations of gingival shade 232 with all three acrylic dental shades received the highest scores (from 6.89 to 7.92). Like shade 232, all the color coordinates of gingival shades 233, 235, 236, and 237 are within the ranges of natural attached gingival color, shade 233 having been chosen by 75.0% of participants as the second‐best option. The combinations of gingival shade 233 with the three dental shades also received good scores (from 6.24 to 6.69), but this was not the case for the gingival–dental combinations achieved with shades 235, 236, and 237, all of which received scores below 5.00.

The three most frequently selected ceramic gingival shades are those with the most natural appearance, making it reasonable to explore the chromatic range between shades 232, 233, and 235 as a basis for expanding the gingival shades available to clinicians, informed by the preferences of the present study's participants. It is worth noting that gingival color coordinates vary in different racial populations [7, 60], and can also be affected by the choice of electronic measurement device: colorimeters and spectroradiometers can produce slightly different results to spectrophotometers. The researchers chose the most widely used spectrophotometer for measuring gingival color, both in vivo and in vitro, due to its high level of reliability (ICC 0.9) [61], although it does have limitations, the most significant of which is “edge loss” [61].

The results obtained demonstrate the usefulness of evaluating combined gingival–dental aesthetics, although they suggest that the range of gingival and dental shades considered should be expanded to better approximate natural color ranges and expand the sample. To date, patients have been involved in selecting the dental shade and only exceptionally in choosing the gingival shade when needed. Prior research has shown that patients like to participate in the gingival–dental rehabilitation process and have their aesthetic preferences on color taken into account [62, 63]. The present results point to the importance of evaluating gingival–dental characteristics conjointly, rather than presenting patients with separate gingival and dental options in order to gain a more realistic understanding of the population's preferences and the resulting chromatic combination prior to prosthesis production.

For each of the three dental shades, statistically significant differences were found between the mean scores for the five gingival–dental combinations (*p* < 0.001 for the three dental shades), which adds to the evidence that gingival appearance plays an important role in aesthetics in dentistry. Statistically significant differences were also identified between the scores for the three gingival–dental combinations with gingival shade 232 (*p* < 0.001) and 233 (*p* = 0.013), whereas the differences did not reach statistical significance for the other gingival shades. The null hypothesis that there are no differences between aesthetic preferences for the various gingival–dental combinations evaluated can therefore be rejected.

Based on the present results, it is reasonable to posit that gingival color has priority when scoring gingival–dental color combinations, and dental color has less weight, since preferences for a certain gingival shade were maintained, irrespective of the dental shade, indicating a stable chromatic hierarchy. This claim needs to be supported by further similar studies that include a greater number of participants, distinct levels of gingival display (in the present study, a wide band of over 3 mm of gingival tissue was displayed), more shades, and a longitudinal design to assess the temporal stability of chromatic preferences. These results could be used to help design a conjoint gingival–dental shade guide that can provide clinicians with a more complete vision of patient opinions by enabling them to evaluate the color of both tissues. Moreover, the importance of gingival color over dental shade should be emphasized and known by dentists, especially in wide smiles.

When comparing the preferences of male and female participants, the only significant difference found was in the mean scores allocated to the 232‐3M1 combination, which women rated higher than men, albeit with a small to moderate effect size. Given that the other comparisons did not produce statistically significant differences, the sample would need to be expanded to reach more definitive conclusions on this point.

Regarding the association between preferences and age, statistically significant differences were found according to age group for the 233‐3M1, 233‐2L1.5, and 235‐1M1 combinations. Younger participants gave lower scores to the first two of these combinations than older participants, perhaps because the dental shades are darker. The opposite occurred for the 235‐1M1 combination, which younger participants scored higher. Again, the sample would need to be expanded to confirm whether preferences vary with age, but the present results provide a basis for future research. The differences identified may be related to young people's greater preference for lighter teeth and tendency to seek whitening treatment which [64, 65], beyond the aesthetic effects, is linked to improved psychosocial characteristics [66, 67, 68, 69, 70]. If we compare sex and age, age seems to have a greater effect on scores for combined gingival–dental aesthetics, although the influence of both factors is low to moderate. On the basis of these results, the null hypothesis that no significant differences exist between aesthetic preferences for the distinct gingival–dental prosthetic combinations according to sex and age can be rejected.

In dentistry, research is lacking on the aesthetics of distinct pink–white color combinations, and it would be very constructive for manufacturers to take this novel perspective on board in the design process. A specific color combination can reinforce the identity of prostheses, creating a sense of visual and aesthetic coherence that increases product recognition and helps patients identify with their appearance. Such research on the interaction between colors and their aesthetic effects can help clinicians take better advantage of color as a visual language, helping patients project an improved aesthetic image, with all the positive psychosocial impacts that implies.

## 5 Conclusions

1. When establishing aesthetic preferences, gingival color takes priority over dental color when both structures are visible in combination.
2. Of the seven shades of ceramic gingival specimen used, aesthetic preferences focused on only two (232 and 233). A substantial majority of participants rated the 232‐1M1 gingival–dental shade combination most highly.
3. Practically no significant differences in the mean scores for gingival–dental color combinations were found in relation to sex. The results showed age to have a greater influence, although the effect of both variables was relatively modest.

## Conflicts of Interest

The authors declare no conflicts of interest.

## Data Availability

The data that support the findings of this study are available on request from the corresponding author. The data are not publicly available due to privacy or ethical restrictions.
